# Retraction Note: High Expression of RIOK2 and NOB1 Predict Human Non-small Cell Lung Cancer Outcomes

**DOI:** 10.1038/s41598-023-32033-5

**Published:** 2023-03-23

**Authors:** Kun Liu, Hong-Lin Chen, Shuo Wang, Ming-Ming Gu, Xin-Ming Chen, Shuang-Long Zhang, Kang-Jun Yu, Qing-Sheng You

**Affiliations:** 1 Department of Cardiothoracic Surgery, Affiliated Hospital of Nantong University, Nantong, 226001 China; 2 Nantong University, Nantong, 226001 China

Retraction of: *Scientific Reports* https://doi.org/10.1038/srep28666, published online 27 June 2016

The Editors have retracted this Article.

After the publication of this Article it was brought to the Editors’ attention that some of the western blot data in Figure 3(b) appear to replicate western blot data in Figure 1B in^1^, which reports an experiment in a different cell type. The Editors reached out to the Authors to request raw data, but were not able to contact any of them, nor to obtain any up to date contact information. Given the concerns about the veracity of the data, the Editors no longer have confidence in the results and conclusions presented in this Article.

Kun Liu, Ming-Ming Gu, Xin-Ming Chen, Shuang-Long Zhang, Kang-Jun Yu, and Qing-Sheng You have not responded to correspondence from the Editors about this retraction. The Editors were not able to obtain current email addresses for Hong-Lin Chen and Shuo Wang.
